# *CPPED1*-targeting microRNA-371a-5p expression in human placenta associates with spontaneous delivery

**DOI:** 10.1371/journal.pone.0234403

**Published:** 2020-06-10

**Authors:** Ravindra Daddali, Marja Ojaniemi, Mikko Hallman, Mika Rämet, Antti M. Haapalainen

**Affiliations:** 1 PEDEGO Research Unit and Medical Research Center Oulu, University of Oulu, Oulu, Finland; 2 Department of Children and Adolescents, Oulu University Hospital, Oulu, Finland; 3 BioMediTech, Institute and Faculty of Medical and Life Sciences, University of Tampere, Tampere, Finland; John Hunter Hospital, AUSTRALIA

## Abstract

MicroRNAs (miRNAs) are important regulators of gene expression, and their expression is associated with many physiological conditions. Here, we investigated potential associations between expression levels of miRNAs in human placenta and the onset of spontaneous term birth. Using RNA sequencing, we identified 54 miRNAs differentially expressed during spontaneous term labor compared to elective term births. Expression levels of 23 miRNAs were upregulated, whereas 31 were downregulated at least 1.5-fold. The upregulated miRNA miR-371a-5p putatively targets *CPPED1*, expression of which decreases during spontaneous birth. We used a luciferase reporter–based assay to test whether a miR-371a-5p mimic affected translation when it bound to the 3′ untranslated region of *CPPED1*. In this setting, the miR-371a-5p mimic resulted in lower luciferase activity, which suggests that miR-371a-5p regulates levels of CPPED1. In conclusion, inversely correlated levels of miR-371a-5p and *CPPED1* suggest a role for both in spontaneous delivery.

## Introduction

Human full-term pregnancy lasts approximately 40 weeks. Known factors that affect the maintenance of pregnancy or initiation of labor include several steroid hormones, peptide hormones, and prostaglandins. Many of these are secreted by the placenta and have effects on implantation, immunomodulation of the mother, cervical remodeling and ripening, and uterine contractions. Maternal immunomodulation in particular is vital to the tolerance of antigens of paternal origin [1–4]. Increased corticotrophin-releasing hormone in maternal plasma and secretion of fetal fibronectin into cervicovaginal fluids are indicators of delivery [5,6]. However, the timing of human labor, particularly the changes at the molecular level resulting in labor, are not currently understood.

Expression of miRNAs are associated with various cellular and physiological functions, including pregnancy, inflammation, infection and immunity, gamete formation, zygote formation, embryogenesis, and fetal development [7,8]. There are three pregnancy-specific miRNA clusters: chromosome 19 miRNA (C19MC) and chromosome 14 miRNA (C14MC) clusters and the miR-371-3 cluster. The miRNAs of these clusters are mainly expressed by the placenta, and expression levels are associated with gestational age [9–11]. The placenta secretes a characteristic pattern of miRNAs into the maternal circulation. Some placenta-secreted miRNAs also traverse into the fetus [12]. Studies have suggested roles for placental miRNAs in both pregnancy and pregnancy-related complications. In particular, C19MC and C14MC miRNAs in the maternal circulation may serve as indicators of preeclampsia and other pregnancy complications [10,13]. Perturbations in placental miRNA and changes in cervical miRNA expression levels at different time points during pregnancy are associated with preeclampsia and preterm birth [14–19]. Recent studies have also demonstrated that miRNA concentration changes are potent biomarkers for predicting preterm labor [15,20–22].

We showed previously that expression levels of calcineurin like phosphoesterase domain containing protein 1 (*CPPED1*) are associated with timing of birth and that spontaneous term placentas have decreased levels of CPPED1 compared to elective term placentas of deliveries without signs or symptoms of labor [23]. Additionally, expression of *CPPED1* is decreased in invasive bladder cancer and overexpression of *CPPED1* delays progression of the cell cycle [24]. Furthermore, *CPPED1* knockdown improves insulin-stimulated glucose uptake in adipocytes [25] and there are type 2 diabetes–associated loci in the *CPPED1* promotor region [26]. Recent studies characterized the antitumor role of CPPED1 by suppressing IL6 expression and secretion levels in cancer patients by activating the STAT3 pathway [27].

CPPED1 dephosphorylates phospho-Ser473 of AKT serine/threonine kinase 1 (AKT1) [24]. AKT1 is part of the phosphatidylinositol 3-kinase (PI3K)/AKT pathway, a conserved intracellular signalling pathway that plays a central role in cellular quiescence, proliferation, cancer, placental development, and fetal growth [28,29]. The PI3K reaction product phosphatidylinositol(3,4,5) triphosphate (PIP3) binds to and recruits pleckstrin homology (PH) domain–containing proteins such as 3-phosphoinositide dependent protein kinase 1 (PDK1), PDK2, and AKT1 to the cell membrane, which activates AKT1 [30]. AKT1 is inactivated by dephosphorylation of two key residues: Thr308 by protein phosphatase 2A (PP2A) and Ser473 by CPPED1 or PH domain and leucine-rich repeat protein phosphatase (PHLPP) [31,32]. FOXO1 transcription factor is an important downstream target of AKT1 [33]. Phosphorylation of FOXO1 by AKT1 results in cytosolic localization of FOXO1 and changes in the transcription of specific genes [34,35]. In the nucleus, FOXO1 and progesterone receptor B are located in the same transcriptional complex [36–38]. Progesterone, the ligand of progesterone receptor, is important for uterine quiescence and, consequently, maintaining pregnancy [2].

In the present study, we performed comparative miRNA expression profiling of placentas from spontaneous term deliveries and elective term births. We found variations in miRNA expression profiles during spontaneous term labor. By comparing the miRNAome data with our previously published human placental proteome [23], we identified a putative labor-associated miRNA:protein pair: miR-371a-5p:CPPED1. MiR-371a-5p is part of the pregnancy-specific miR-371-3 cluster. We found evidence suggesting that miR-371a-5p regulates *CPPED1* mRNA during spontaneous delivery.

## Materials and methods

### Placental tissue samples

Human placental tissue samples were collected at Oulu University Hospital as described previously [39] and included samples from the basal and chorionic plates of the placenta. The ethics committee of Oulu University approved the study, and all mothers provided written informed consent. All experiments were performed in accordance with relevant guidelines and regulations. Mothers who delivered electively by caesarean section had no signs and symptoms of labor. Consequently, for our miRNAomic analyses we could use these elective term deliveries as a control to compare with spontaneous term labors to identify changes associated with the initiation of spontaneous labor. We also performed qPCR analysis of *CPPED1* expression in spontaneous preterm versus spontaneous term labor to investigate whether expression changes were associated with the length of pregnancy.

### MiRNAomes of human placentas

The miRNAomic study included placental samples from the basal plate (*n* = 6 per group) collected after either spontaneous term (gestational age from 40 weeks + 0 days to 41 weeks + 3 days) or elective caesarean term (gestational age from 38 weeks + 0 days to 42 weeks + 0 days) birth. Births with pregnancy- or labor-associated complications such as placental abruption, polyhydramnios, and preeclampsia were excluded. MiRNAs were isolated with the NucleoSpin miRNA kit (Macherey-Nagel). RNA sample quality was good, as determined with the Fragment Analyzer (Advanced Analytical Technologies). MiRNA library preparation and HiSeq 2500 sequencing were done at the Finnish Functional Genomics Centre in Finland. Sequencing data were analyzed by the Bioinformatics Unit at the Turku Centre for Biotechnology and Biocenter Finland. A fold-change of 1.5 and *p* value of 0.05 were set as threshold values when filtering differentially expressed miRNAs.

### Quantitative PCR of *hsa-miR-371a-5p*

MicroRNAs were isolated from human placentas with the NucleoSpin miRNA kit (Macherey-Nagel), and RNA quality was determined with the Agilent 2100 Bionalyzer system at the Biocenter Oulu Sequencing Center in Finland. First-strand cDNA synthesis was done with the miRCURY LNA RT Kit (Qiagen). The following samples were included in qRT-PCR: spontaneous term birth (*n* = 19) and elective term birth (*n* = 14). Gestational ages were 38 weeks + 6 days to 41 weeks + 6 days for spontaneous term birth and 38 weeks + 0 days to 42 weeks + 0 days for elective term birth. qPCR was done with the miRCURY LNA miRNA PCR Assay kit (Qiagen) on a LightCycler®96 (Roche). Validated and optimized PCR primers hsa-miR-371a-5p (YP00204493) and hsa-miR-103a-3p (YP00204063) were obtained from Qiagen. *hsa-miR-103a-3p* mRNA was used as a reference gene. All samples were measured in triplicate and normalized in accordance with the ΔΔC_t_ method. Melting curve analysis and agarose gel electrophoresis indicated a single and specific PCR product. Statistical analyses were done with SPSS Statistics 20.0 (IBM Corporation). Significant differences in expression levels were identified by nonparametric Mann–Whitney *U* test.

### Quantitative PCR of *CPPED1*

**Fig 1:**
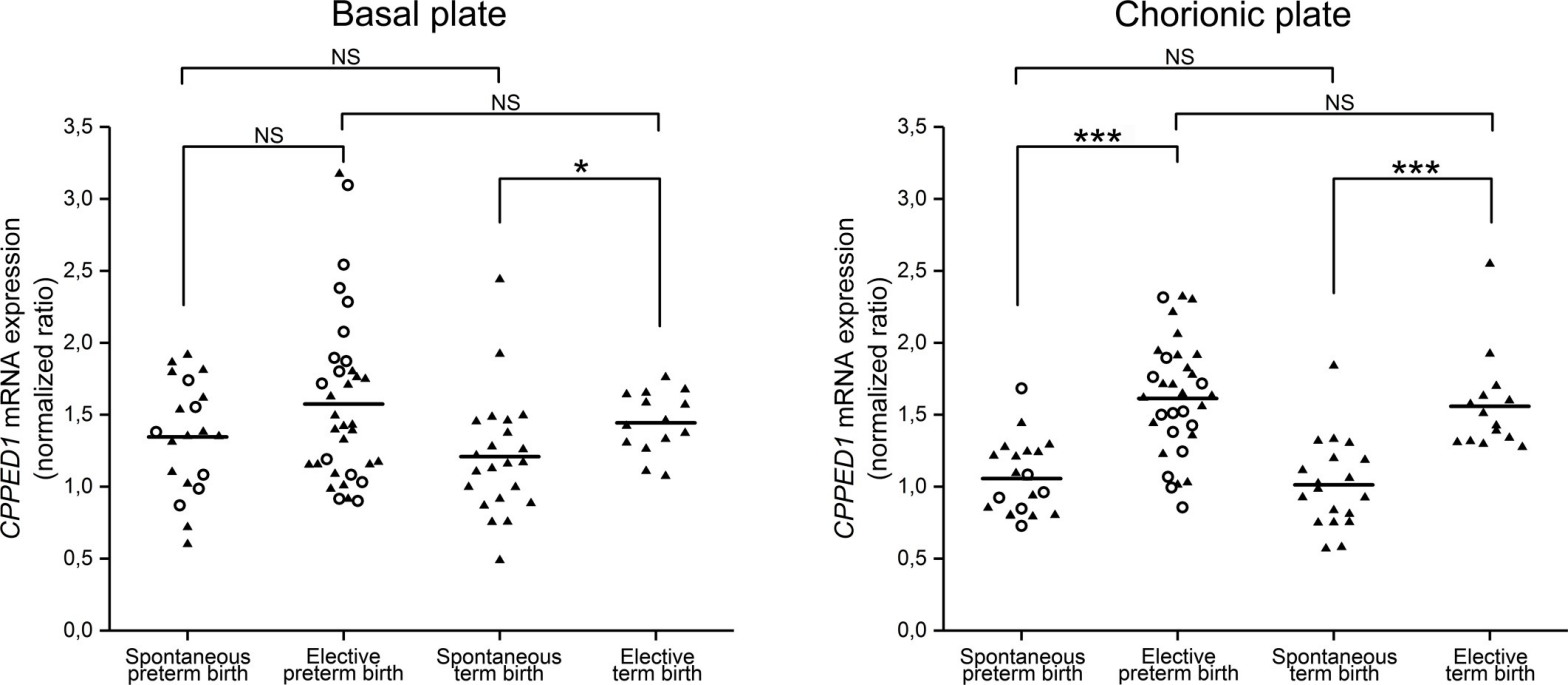
*CPPED1* mRNA levels in preterm and term placentas. Normalized expression levels of *CPPED1* in human preterm and term placentas to compare spontaneous and elective deliveries. *CPPED1* expression was decreased during spontaneous preterm birth in the chorionic plate (right panel) but not in the basal plate (left panel). At spontaneous term birth, *CPPED1* mRNA levels decreased in both basal and chorionic plates. There was no difference in *CPPED1* expression in placentas from preterm and term spontaneous and elective deliveries. Differences were analyzed with nonparametric Kruskall–Wallis test. Horizontal line denotes the median of each group. Statistically significant changes are indicated by one (*p* < 0.05) and three (*p* < 0.001) asterisks; NS, not significant. Preeclampsia and intra-amniotic inflammation cases are indicated by an open circle.

qRT-PCR analysis included samples of the following placentas: basal plate of spontaneous preterm birth (*n* = 20), chorionic plate of spontaneous preterm birth (*n* = 20), basal plate of elective preterm birth (*n* = 34), chorionic plate of elective preterm birth (*n* = 33), basal plate of spontaneous term birth (*n* = 22), chorionic plate of spontaneous term birth (*n* = 19), basal plate of elective term birth (*n* = 14), and chorionic plate of elective term birth (*n* = 14). Gestational ages were 25 weeks + 2 days to 36 weeks + 6 days for spontaneous preterm labor, 25 weeks + 1 day to 36 weeks + 6 days for elective preterm birth, 38 weeks + 6 days to 41 weeks + 6 days for spontaneous term birth, and 38 weeks + 1 day to 42 weeks + 0 days for elective term birth. Only the spontaneous preterm and elective preterm groups contained cases of preeclampsia and intra-amniotic inflammation (Fig 1). RNeasy Micro Kit (Qiagen) was used for RNA isolation. cDNA synthesis and quantitative PCR (qPCR) were done as described previously [23,39]. Briefly, qPCR was done as an intron-spanning assay with the LightCycler®96 (Roche). Cytochrome c1 (CYC1) mRNA was used as a reference gene. All samples were measured in triplicate and normalized in accordance with the ΔΔC_t_ method. Statistical analyses were conducted with SPSS Statistics 20.0 (IBM Corporation). Significant differences in expression levels were identified by nonparametric Kruskall–Wallis test, in which significance values were adjusted by the Bonferroni correction for multiple tests.

### Cloning of 3′ untranslated region (UTR) of *CPPED1* into pmirGLO vector

The 3′ UTR of *CPPED1* was amplified by PCR with two primer pairs: 5′-cacttgctagcCGCTCCTTCCCGTTCCCG-3′ and 5′-catctgtcgacTAATTTACAAAGAAAAGAGATTTAATAGACTC-3′ for the full-length 3′UTR (WT), and 5′-cacttgctagcCGCTCCTTCCCGTTCCCG-3′ and 5′-catctgtcgacCAGTTCCGCATGGCTGGG-3′ for the 3′ UTR lacking the last 33 base pairs (Mut). Mismatches to the 3′ UTR region are indicated by lowercase, while introduced *Nhe*I and *Sal*I sites at the 5′ and 3′ ends, respectively, of the PCR products are underlined. The PCR products WT (5087 bp) and Mut (5054 bp) were first subcloned into pJET1.2 blunt cloning vector (Thermo Scientific). Nucleotide sequences of the inserts were verified. The introduced *Nhe*I and *Sal*I restriction sites allowed for release of the insert and ligation into the similarly digested pmirGLO vector (Promega), resulting in the full-length 3′ UTR of *CPPED1* (WT+pmirGLO) and a shorter variant of the 3′ UTR of *CPPED1* (Mut+pmirGLO) in the pmirGLO vector.

### Luciferase reporter assay

HEK-293T cells were used in the luciferase reporter assay because of their high transfection efficiency. HEK-293T cells were cultured in DMEM (Invitrogen Life Technologies) supplemented with 10% fetal bovine serum (Invitrogen Life Technologies), 100 U/ml penicillin, and 100 mg/ml streptomycin. Subculturing was performed with 0.05% trypsin/0.02% EDTA. Before transfection, cells were plated onto 96-well plates and allowed to reach approximately 90% confluence. On the day of transfection, HEK-293T cells were cotransfected with 100 ng of plasmid DNA (WT+pmirGLO or Mut+pmirGLO)–Lipofectamine3000 (Invitrogen Life Technologies) and miRNA (mimic or mimic negative control)-Lipofectamine3000 complexes, resulting in 10 nM miRNA mimic or negative control per well. MiRNA mimics were hsa-miR-371a-5p (MC12791, Ambion), hsa-miR-520d-5p (MC12934, Ambion), and hsa-miR-524-5p (MC10753, Ambion). mirVana™ miRNA Mimic, Negative Control #1 (Ambion) was used as a negative control. Transfections were performed in triplicate in two independent experiments. Cells were lysed 24 hours post-transfection, and luciferase reporter assays were performed with the Promega Dual-Luciferase Reporter Assay System (Promega). *Firefly* and *Renilla* luciferase activities were measured, and background signal was subtracted for each transfection. *Firefly* (experimental reporter) and *Renilla* (control reporter) luciferase ratios were calculated and compared with negative controls, which were set to 1.00. Significant differences were estimated with the nonparametric Mann–Whitney *U* test (*n* = 6 per group).

## Results

### MiRNAomics of human placenta identifies miRNAs up- and downregulated at spontaneous term labor

MiRNAs regulate many physiological processes by affecting mRNA and protein levels [41]. To investigate whether expression levels of placental miRNAs are associated with spontaneous term labor, we characterized the miRNAomes of human placentas after spontaneous delivery (*n* = 6) and elective birth (*n* = 6). There were no signs or symptoms of labor in the cases of elective birth. miRNAomic comparisons of these two placental phenotypes revealed that 23 and 31 miRNAs were up- and downregulated, respectively, during spontaneous term labor (Table 1). miR-323b-3p is a member of placenta-specific C14MC, while miR-373-3p, miR-371a-5p, miR-371b-3p, miR-372-3p, and miR-372-5p are members of the placenta-specific miR-371-3 cluster (Table 1). The genes of the rest of the differentially regulated miRNAs are located in various different chromosomes. MiRNAs of C14MC are predominantly expressed during the first trimester of pregnancy, and C19MC members, together with members of the miR-371-3 cluster, are highly expressed toward the end of pregnancy [10,18]. Our data indicate that expression of selected miRNAs changes during the onset of labor.

**Table 1:** Up- and downregulated human placenta miRNAs at spontaneous term labor. MiRNAs isolated from human placenta after spontaneous term labor were compared with miRNAs of placentas obtained from elective deliveries by caesarean section without signs or symptoms of labor. Thresholds used in filtering differentially expressed miRNAs were fold-change (FC) more or less than 1.5 and *p* value < 0.05. MiRNAs were ranked based on both *t*-test *p* value and FC. In the average rank column, the value 1 is the strongest differentially expressed miRNA. Up- and downregulated miRNAs are shown in red and green, respectively. Chromosomal locations of miRNAs belonging to either C14MC or miR-371-3 cluster are highlighted in orange.

MicroRNA	Average rank	Fold-change	*p* Value	Chromosomal location
hsa-miR-373-3p	2	2.61	0.001	19q13.42
hsa-miR-371a-5p	6	2.47	0.003	19q13.42
hsa-miR-371b-3p	7	2.34	0.003	19q13.42
hsa-miR-372-3p	11	2.16	0.007	19q13.42
hsa-miR-323b-3p	15	1.83	0.008	14q32.31
hsa-miR-760	23	1.70	0.007	1p22.1
hsa-miR-1254	26	1.63	0.007	NA
hsa-miR-551a	28	1.67	0.009	1p36.32
hsa-miR-184	29	1.66	0.012	15q25.1
hsa-miR-6511b-3p	30	1.70	0.017	16p13.3
hsa-miR-4707-5p	38	1.60	0.022	14q11.2
hsa-miR-372-5p	39	1.62	0.026	19q13.42
hsa-miR-509-3p	40	1.62	0.025	Xq27.3
hsa-miR-6779-5p	44	1.54	0.018	17q12
hsa-miR-504-5p	46	1.68	0.040	Xq26.3
hsa-miR-99b-3p	48	1.49	0.014	19q13.41
hsa-miR-3140-3p	50	1.49	0.016	4q31.3
hsa-miR-133a-3p	52	1.52	0.021	18q11.2
hsa-miR-205-5p	54	1.60	0.036	1q32.2
hsa-miR-877-5p	59	1.49	0.023	6p21.33
hsa-miR-551b-5p	67	1.53	0.044	3q26.2
hsa-miR-449c-3p	71	1.46	0.033	5q11.2
hsa-miR-4665-3p	73	1.47	0.038	9p24.1
hsa-miR-4732-5p	1	-3.28	0.001	17q11.2
hsa-miR-6743-5p	4	-2.50	0.001	11p15.5
hsa-miR-1272	5	-2.86	0.005	15q22.31
hsa-miR-6765-3p	8	-2.35	0.004	14q32.33
hsa-miR-6872-3p	9	-2.08	0.004	3p21.31
hsa-miR-135a-5p	13	-4.79	0.026	3p21.2
hsa-miR-3144-3p	14	-1.97	0.009	6q22.31
hsa-miR-5100	16	-1.86	0.010	10q11.21
hsa-miR-6865-5p	17	-1.79	0.007	17p13.2
hsa-miR-501-5p	18	-1.99	0.013	Xp11.23
hsa-miR-5196-5p	19	-1.82	0.010	19q13.12
hsa-miR-2277-3p	20	-2.06	0.016	5q15
hsa-miR-1260a	21	-1.68	0.002	14q24.3
hsa-miR-1909-3p	22	-1.84	0.014	19p13.3
hsa-miR-4286	24	-1.70	0.011	8p23.1
hsa-let-7g-3p	27	-1.80	0.020	3p21.2
hsa-miR-1292-5p	34	-1.68	0.027	20p13
hsa-miR-4784	35	-1.63	0.026	2q21.1
hsa-miR-4304	37	-1.53	0.013	12q24.31
hsa-miR-3928-5p	41	-1.70	0.038	22q12.2
hsa-miR-4664-5p	42	-1.53	0.014	8q24.3
hsa-miR-451a	45	-1.62	0.033	17q11.2
hsa-miR-6069	47	-1.46	0.008	22q12.3
hsa-miR-3614-3p	49	-1.52	0.018	17q22
hsa-miR-3117-3p	53	-1.54	0.026	1p31.3
hsa-miR-7850-5p	57	-1.48	0.020	19p13.3
hsa-miR-3922-5p	58	-1.55	0.036	12q23.3
hsa-miR-1908-3p	61	-1.60	0.044	11q12.2
hsa-miR-6763-5p	62	-1.58	0.042	12q24.33
hsa-miR-6071	63	-1.50	0.027	2p11.2
hsa-miR-7977	64	-1.46	0.022	3q26.32

### Functional annotation of miRNA targets shows enrichment of cancer- and PI3K-AKT–related pathways

A single miRNA can have hundreds of mRNA targets. To find out which pathways are enriched for targets of the miRNAs that we determined were up- and downregulated (Table 1) during spontaneous labor, we obtained validated targets of the miRNAs from the Micro-RNA-Target Database (miRTarBase) [42] (Table 2). These targets have been validated experimentally by reporter assay, western blot, or qPCR, resulting in strong evidence for the miRNA:mRNA pairing [42]. Next, we included all of the identified targets (Table 2) in an analysis performed with the DAVID functional annotation tool [43]. Within the list of identified KEGG terms, cancer-related pathways were overrepresented (Table 3). Moreover, functional annotation clustering revealed that the most significant cluster, with an enrichment score of 16.05, comprised hsa05200:Pathways in cancer, hsa05215:Prostate cancer, hsa04151:PI3K-Akt signaling pathway, and hsa04510:Focal adhesion (Table 3). Interestingly, the hsa04068:FoxO signaling pathway was also one of the targeted pathways. We also separately analyzed the miRNA targets of up- and downregulated miRNAs (Table 2) with the DAVID functional annotation tool; these results are shown in Table 4. Cancer-related pathways were also overrepresented in this analysis, and similar KEGG terms were obtained for the targets of both up- and downregulated miRNAs (Table 4).

**Table 2:** Human placental miRNAs and their experimentally validated targets. MiRNAs that were either up- (red) or down- (green) regulated at the onset of labor and have validated targets are shown. Listed miRNA targets were obtained with miRTarBase software. All miRNA–target interactions have been validated by reporter assay, western blot, and/or qPCR. miRNAs are in the same order as listed in Table 1.

MicroRNA	Validated targets
hsa-miR-373-3p	BTG1, CD44, CSDC2, DKK1, IRF9, JAK1, LATS2, LEFTY1, LEFTY2, MBD2, MRE11, MTOR, MYC, NFIB, PIK3CA, RABEP1, RAD23B, RAD52, RASSF1, RECK, SIRT1, TGFBR2, TNFAIP1, TXNIP, VEGFA, XPA
hsa-miR-371a-5p	CDH1, HSP90AA1, PRPF4B, PTENP1, SOX2
hsa-miR-372-3p	ATAD2, BTG1, CCNA1, CDK2, CDKN1A, DKK1, ERBB4, KLF13, LATS2, LEFTY1, MBNL2, NFIB, NR4A2, PHLPP2, RHOC, TGFBR2, TNFAIP1, TRPS1, TXNIP, WEE1, VEGFA
hsa-miR-760	CSNK2A1, HIST1H2AD, HIST1H3D
hsa-miR-1254	CCAR1
hsa-miR-551a	CKB, PTP4A3
hsa-miR-184	AGO2, AKT1, AKT2, BCL2, BIN3, EZR, GAS1, INPPL1, MYC, NFATC2, PDGFB, PKM, PLPP3, PPP1R13L, PRKCB, SND1, SOX7, TNFAIP2, ZFPM2
hsa-miR-509-3p	CFTR, MAP3K8, NTRK3, XIAP, YAP1
hsa-miR-504-5p	BAX, BBC3, CDK6, DRD1, FAS, GADD45A, MDM2, TCEAL1, TFF1, TP53, TP53I3, VEGFA
hsa-miR-99b-3p	GSK3B
hsa-miR-133a-3p	CDC42, HCN4, UCP2, KRT7, CACNA1C, HCN2, CASP9, KCNQ1, FSCN1, KCNH2, TAGLN2, LASP1, PNP, MSN, EGFR, VKORC1, PRDM16, ARPC5, FTL, EGFL7, VEGFA, PIK3R2, RGS3, COL1A1, SP1, BCL2L1, MCL1, RFFL, IGF1R, UBA2, ANXA2, MMP14, SNX30, SGMS2, PDLIM5, IGF1, ZEB1, CTGF, LDLRAP1, NR2C2, MEG3, AFTPH, ERBB2, FOXl2, SOX4, RBPJ, NGFR, GSTP1
hsa-miR-205-5p	LRP1, ZEB1, DDX5, INPPL1, MED1, E2F5, E2F1, ERBB3, ZEB2, PRKCE, VEGFA, SIGMAR1, IL24, IL32, EGLN2, TP73, CYR61, CTGF, ERBB2, LAMC1, LRRK2, YES1, SRC, BCL2, SMAD1, SMAD4, YY1, PTPRM, AR, BCL6, ACSL4, ITGA5, ACSL1, HMGB3, PTEN, ESRRG, PHLPP2, KCNJ10, EZR, LMNA, RUNX2, HMGB1, PRDX2, SMAD2, UVRAG, CENPF, SATB2, CCNJ
hsa-miR-135a-5p	JAK2, NR3C2, APC, HOXA10, MYC, SMAD5, BMPR2, STAT6, MTSS1, ROCK1, VLDLR, TXNIP, IRS2, APC, HTR1A, SLC6A4, SIAH1, BCL2, KLF8, ESRRA, RUNX2, ROCK2, FOXO1, PHLPP2, E2F1, DAPK2, KLF4, PTPRD, PTK2, CEBPD, PPM1E, MMP11, RBAK, EGFR
hsa-miR-501-5p	AKT2, DKK1, DNAJB14, INPPL1, LAMTOR5, NFATC2, NKD1
hsa-let-7g-3p	CCL2, CCL5, MYBPC3
hsa-miR-1292-5p	SOX4
hsa-miR-4304	AKT2, INPPL1, NFATC2
hsa-miR-451a	ABCB1, ADAM10, AKT1, BCL2, CAB39, CDKN2D, CPNE3, DCBLD2, FRZB, IKBKB, IL6, IL6R, MAP3K1, MAPK1, MIF, MMP2, MMP9, MYC, OSR1, OXTR, PKD1, RAB14, RAB5A, ROR2, TMED7, TSC1

**Table 3:** Functional annotation of miRNA targets associated with spontaneous term birth. Clustering was performed by DAVID analysis [43] according to KEGG pathways and used all of the identified miRNA targets listed in Table 2. KEGG terms with false discovery rate (FDR)–adjusted *p* values of <0.05 are shown. In functional annotation clustering, KEGG terms highlighted in orange comprised the most significant cluster, with an enrichment score of 16.05.

KEGG term	Number of genes involved in the KEGG term	*p* Value	Benjamini-Hochberg–corrected *p* value	FDR-adjusted *p* value
hsa05200:Pathways in cancer	50	6.30 × 10^−24^	1.27 × 10^−21^	7.90 × 10^−21^
hsa05215:Prostate cancer	24	1.07 × 10^−18^	1.08 × 10^−16^	1.34 × 10^−15^
hsa05161:Hepatitis B	28	1.05 × 10^−17^	7.05 × 10^−16^	1.32 × 10^−14^
hsa05205:Proteoglycans in cancer	31	7.33 × 10^−17^	5.55 × 10^−15^	1.44 × 10^−13^
hsa05212:Pancreatic cancer	19	2.61 × 10^−15^	1.03 × 10^−13^	3.21 × 10^−12^
hsa05206:MicroRNAs in cancer	33	3.87 × 10^−14^	1.29 × 10^−12^	4.85 × 10^−11^
hsa04151:PI3K-Akt signaling pathway	36	4.07 × 10^−14^	1.17 × 10^−12^	5.10 × 10^−11^
hsa05219:Bladder cancer	15	1.44 × 10^−13^	3.61 × 10^−12^	1.80 × 10^−10^
hsa04068:FoxO signaling pathway	23	2.33 × 10^−13^	5.21 × 10^−12^	2.93 × 10^−10^
hsa05214:Glioma	17	7.59 × 10^−13^	1.52 × 10^−11^	9.52 × 10^−10^
hsa05218:Melanoma	16	4.43 × 10^−11^	8.09 × 10^−10^	5.55 × 10^−08^
hsa05222:Small cell lung cancer	17	6.19 × 10^−11^	1.04 × 10^−09^	7.76 × 10^−08^
hsa05210:Colorectal cancer	15	7.80 × 10^−11^	1.21 × 10^−09^	9.79 × 10^−08^
hsa05213:Endometrial cancer	14	9.58 × 10^−11^	1.37 × 10^−09^	1.20 × 10^−07^
hsa04510:Focal adhesion	24	2.35 × 10^−10^	3.15 × 10^−09^	2.95 × 10^−07^
hsa04115:p53 signaling pathway	15	2.38 × 10^−10^	2.98 × 10^−09^	2.98 × 10^−07^
hsa04066:HIF-1 signaling pathway	17	4.19 × 10^−10^	4.95 × 10^−09^	5.25 × 10^−07^
hsa05220:Chronic myeloid leukemia	15	6.57 × 10^−10^	7.33 × 10^−09^	8.24 × 10^−07^
hsa04012:ErbB signaling pathway	16	9.26 × 10^−10^	9.80 × 10^−09^	1.16 × 10^−06^
hsa04550:Signaling pathways regulating pluripotency of stem cells	19	2.55 × 10^−09^	2.56 × 10^−08^	3.20 × 10^−06^
hsa05223:Non-small cell lung cancer	13	3.56 × 10^−09^	3.41 × 10^−08^	4.46 × 10^−06^
hsa05202:Transcriptional misregulation in cancer	20	7.18 × 10^−09^	6.56 × 10^−08^	9.01 × 10^−06^
hsa05230:Central carbon metabolism in cancer	13	1.78 × 10^−08^	1.56 × 10^−07^	2.24 × 10^−05^
hsa05162:Measles	17	5.41 × 10^−08^	4.53 × 10^−07^	6.79 × 10^−05^
hsa04520:Adherens junction	13	6.05 × 10^−08^	4.86 × 10^−07^	7.59 × 10^−05^
hsa04150:mTOR signaling pathway	12	6.35 × 10^−08^	4.91 × 10^−07^	7.96 × 10^−05^
hsa04370:VEGF signaling pathway	12	1.10 × 10^−07^	8.17 × 10^−07^	1.38 × 10^−04^
hsa04210:Apoptosis	12	1.31 × 10^−07^	9.39 × 10^−07^	1.64 × 10^−04^
hsa05166:HTLV-I infection	22	3.12 × 10^−07^	2.16 × 10^−06^	3.92 × 10^−04^
hsa04919:Thyroid hormone signaling pathway	15	3.31 × 10^−07^	2.22 × 10^−06^	4.16 × 10^−04^
hsa04350:TGF-beta signaling pathway	13	4.12 × 10^−07^	2.67 × 10^−06^	5.17 × 10^−04^
hsa04722:Neurotrophin signaling pathway	15	5.64 × 10^−07^	3.54 × 10^−06^	7.08 × 10^−04^
hsa05169:Epstein-Barr virus infection	15	6.92 × 10^−07^	4.22 × 10^−06^	8.68 × 10^−04^
hsa04152:AMPK signaling pathway	15	7.66 × 10^−07^	4.53 × 10^−06^	9.61 × 10^−04^
hsa04110:Cell cycle	15	8.46 × 10^−07^	4.86 × 10^−06^	1.06 × 10^−03^
hsa04390:Hippo signaling pathway	16	1.77 × 10^−06^	9.87 × 10^−06^	2.22 × 10^−03^
hsa04071:Sphingolipid signaling pathway	14	3.40 × 10^−06^	1.85 × 10^−05^	4.26 × 10^−03^
hsa04668:TNF signaling pathway	13	5.71 × 10^−06^	3.02 × 10^−05^	7.16 × 10^−03^
hsa05164:Influenza A	16	1.03 × 10^−05^	5.32 × 10^−05^	1.30 × 10^−03^
hsa04930:Type II diabetes mellitus	9	1.17 × 10^−05^	5.90 × 10^−05^	1.47 × 10^−02^
hsa05203:Viral carcinogenesis	17	1.83 × 10^−05^	8.98 × 10^−05^	2.30 × 10^−02^
hsa05231:Choline metabolism in cancer	12	1.88 × 10^−05^	9.01 × 10^−05^	2.36 × 10^−02^
hsa04666:Fc gamma R-mediated phagocytosis	11	2.08 × 10^−05^	9.73 × 10^−05^	2.61 × 10^−02^
hsa05142:Chagas disease (American trypanosomiasis)	12	2.49 × 10^−05^	1.14 × 10^−05^	3.12 × 10^−02^

**Table 4:** Functional annotation of miRNA targets of up- and downregulated miRNAs associated with spontaneous term birth. Targets associated with either up- or downregulated miRNAs of Table 2 analyzed separately by DAVID analysis [43] according to KEGG pathways. Results for targets of up- and downregulated miRNAs are shown in red and green, respectively. KEGG terms with false discovery rate (FDR)–adjusted *p* values of < 0.05 are shown.

KEGG Term	Number of genes involved in the KEGG term	*p* Value	Benjamini-Hochberg–corrected *p* value	FDR-adjusted *p* value
hsa05200:Pathways in cancer	39	4.61 × 10^−19^	8.99 × 10^−17^	5.75 × 10^−16^
hsa05215:Prostate cancer	21	1.75 × 10^−17^	1.71 × 10^−15^	2.18 × 10^−14^
hsa05212:Pancreatic cancer	17	9.64 × 10^−15^	6.28 × 10^−13^	1.21 × 10^−11^
hsa05205:Proteoglycans in cancer	25	3.92 × 10^−14^	1.91 × 10^−12^	4.89 × 10^−11^
hsa05214:Glioma	16	1.96 × 10^−13^	7.64 × 10^−12^	2.45 × 10^−10^
hsa05161:Hepatitis B	21	4.66 × 10^−13^	1.51 × 10^−11^	5.82 × 10^−10^
hsa04151:PI3K-Akt signaling pathway	29	4.48 × 10^−12^	1.25 × 10^−10^	5.59 × 10^−09^
hsa05218:Melanoma	15	1.31 × 10^−11^	3.20 × 10^−10^	1.64 × 10^−08^
hsa05206:MicroRNAs in cancer	26	1.52 × 10^−11^	3.29 × 10^−10^	1.89 × 10^−08^
hsa04115:p53 signaling pathway	14	9.16 × 10^−11^	1.79 × 10^−09^	1.14 × 10^−07^
hsa05222:Small cell lung cancer	15	1.69 × 10^−10^	2.99 × 10^−09^	2.10 × 10^−07^
hsa05210:Colorectal cancer	13	5.20 × 10^−10^	8.45 × 10^−09^	6.49 × 10^−07^
hsa05213:Endometrial cancer	12	1.04 × 10^−09^	1.56 × 10^−08^	1.29 × 10^−06^
hsa04068:FoxO signaling pathway	17	1.10 × 10^−09^	1.54 × 10^−08^	1.38 × 10^−06^
hsa05219:Bladder cancer	11	1.38 × 10^−09^	1.79 × 10^−08^	1.72 × 10^−06^
hsa04510:Focal adhesion	20	2.31 × 10^−09^	2.81 × 10^−08^	2.88 × 10^−06^
hsa05223:Non-small cell lung cancer	12	2.41 × 10^−09^	2.76 × 10^−08^	3.01 × 10^−06^
hsa04012:ErbB signaling pathway	14	2.75 × 10^−09^	2.98 × 10^−08^	3.43 × 10^−06^
hsa05220:Chronic myeloid leukemia	13	3.19 × 10^−09^	3.28 × 10^−08^	3.98 × 10^−06^
hsa04066:HIF-1 signaling pathway	14	9.50 × 10^−09^	9.26 × 10^−08^	1.19 × 10^−05^
hsa05230:Central carbon metabolism in cancer	12	1.07 × 10^−08^	9.92 × 10^−08^	1.33 × 10^−05^
hsa04520:Adherens junction	12	3.32 × 10^−08^	2.94 × 10^−07^	4.14 × 10^−05^
hsa05162:Measles	15	6.69 × 10^−08^	5.67 × 10^−07^	8.35 × 10^−05^
hsa04210:Apoptosis	11	9.70 × 10^−08^	7.88 × 10^−07^	1.21 × 10^−04^
hsa05169:Epstein-Barr virus infection	14	1.76 × 10^−07^	1.37 × 10^−06^	2.20 × 10^−04^
hsa04110:Cell cycle	14	2.13 × 10^−07^	1.60 × 10^−06^	2.66 × 10^−04^
hsa04919:Thyroid hormone signaling pathway	13	6.78 × 10^−07^	4.89 × 10^−06^	8.46 × 10^−04^
hsa04370:VEGF signaling pathway	10	9.61 × 10^−07^	6.69 × 10^−06^	1.20 × 10^−03^
hsa05202:Transcriptional misregulation in cancer	15	1.14 × 10^−06^	7.65 × 10^−06^	1.42 × 10^−03^
hsa05166:HTLV-I infection	18	1.82 × 10^−06^	1.18 × 10^−05^	2.27 × 10^−03^
hsa04390:Hippo signaling pathway	14	2.09 × 10^−06^	1.31 × 10^−05^	2.61 × 10^−03^
hsa05203:Viral carcinogenesis	16	2.59 × 10^−06^	1.58 × 10^−05^	3.23 × 10^−03^
hsa04550:Signaling pathways regulating pluripotency of stem cells	13	5.53 × 10^−06^	3.27 × 10^−05^	6.90 × 10^−03^
hsa04722:Neurotrophin signaling pathway	12	7.27 × 10^−06^	4.17 × 10^−05^	9.08 × 10^−03^
hsa04666:Fc gamma R-mediated phagocytosis	10	1.45 × 10^−05^	8.10 × 10^−05^	1.81 × 10^−02^
hsa05161:Hepatitis B	13	1.77 × 10^−10^	2.43 × 10^−08^	2.08 × 10^−07^
hsa05200:Pathways in cancer	17	3.97 × 10^−09^	2.72 × 10^−07^	4.67 × 10^−06^
hsa05219:Bladder cancer	7	3.13 × 10^−07^	1.43 × 10^−05^	3.68 × 10^−04^
hsa05215:Prostate cancer	8	2.12 × 10^−06^	7.26 × 10^−05^	2.49 × 10^−03^
hsa04151:PI3K-Akt signaling pathway	13	2.84 × 10^−06^	7.77 × 10^−05^	3.34 × 10^−03^
hsa04068:FoxO signaling pathway	9	3.29 × 10^−06^	7.52 × 10^−05^	3.87 × 10^−03^
hsa04062:Chemokine signaling pathway	10	4.30 × 10^−06^	8.41 × 10^−05^	5.05 × 10^−03^
hsa04550:Signaling pathways regulating pluripotency of stem cells	9	4.57 × 10^−06^	7.83 × 10^−05^	5.38 × 10^−03^
hsa05205:Proteoglycans in cancer	10	7.78 × 10^−06^	1.18 × 10^−04^	9.15 × 10^−03^
hsa04668:TNF signaling pathway	8	7.88 × 10^−06^	1.08 × 10^−04^	9.27 × 10^−03^
hsa05206:MicroRNAs in cancer	11	2.12 × 10^−05^	2.64 × 10^−04^	2.49 × 10^−02^
hsa05222:Small cell lung cancer	7	2.41 × 10^−05^	2.76 × 10^−04^	2.84 × 10^−02^

### Comparison of human placental proteomics and miRNAomics identifies CPPED1 as a common denominator

We previously determined the proteomics of the human placenta [23]. In this prior study, we identified ten proteins that were up- or downregulated in the placenta at spontaneous term delivery. None of these ten proteins were among the validated targets of the miRNAs (Table 2) that we identified in the placenta at spontaneous term delivery. To identify putative novel targets for the miRNAs (Table 1), we investigated whether there was any overlap between the proteomics [23] and computationally predicted targets of miRNAs of miRNAomics (Table 5). We found that our 54 miRNAs were computationally predicted to bind to 27,708 targets in total, based only on the 8mers/canonical sites found in the 3′ UTRs of the targets. We included these 27,708 predicted miRNA targets and ten proteins identified by proteomics (actin cytoplasmic 1 [ACTB], β‐2‐microglobulin, keratin type II cytoskeletal 8, keratin type II cytoskeletal 19, α‐2‐macroglobulin, CPPED1, cytochrome b5, hemoglobin subunit γ‐2, peroxiredoxin‐2, and plasminogen activator inhibitor 2) in the comparison and identified only three miRNA:target pairs in the basal plate of the placenta: miR-371a-5p:CPPED1, miR-3614-3p:ACTB, and miR-6872-3p:ACTB. During spontaneous term delivery, miR-371a-5p was upregulated (Table 1) but CPPED1 was downregulated [23], suggesting potential post-transcriptional regulation of CPPED1 by miR-371a-5p. By contrast, levels of miR-3614-3p and miR-6872-3p were downregulated and ACTB upregulated during spontaneous term delivery.

**Table 5:** Number of predicted targets of miRNAs in the placental miRNAome. Predicted targets for each miRNA were determined by analyzing an online database for miRNA target prediction and functional annotations (miRDB) [44,45]. The target prediction score was set between 50 and 100. Because of the high number of predicted targets for each miRNA, only the number of targets are shown. Results for targets of up- and downregulated miRNAs are shown in red and green, respectively. miRNAs are listed in the same order as in Table 1.

MicroRNA	Number of predicted targets
hsa-miR-373-3p	899
hsa-miR-371a-5p	876
hsa-miR-371b-3p	190
hsa-miR-372-3p	902
hsa-miR-323b-3p	323
hsa-miR-760	690
hsa-miR-1254	NA
hsa-miR-551a	21
hsa-miR-184	70
hsa-miR-6511b-3p	341
hsa-miR-4707-5p	60
hsa-miR-372-5p	1050
hsa-miR-509-3p	282
hsa-miR-6779-5p	1225
hsa-miR-504-5p	317
hsa-miR-99b-3p	54
hsa-miR-3140-3p	1049
hsa-miR-133a-3p	654
hsa-miR-205-5p	737
hsa-miR-877-5p	354
hsa-miR-551b-5p	1632
hsa-miR-449c-3p	235
hsa-miR-4665-3p	7
hsa-miR-4732-5p	186
hsa-miR-6743-5p	339
hsa-miR-1272	432
hsa-miR-6765-3p	536
hsa-miR-6872-3p	344
hsa-miR-135a-5p	804
hsa-miR-3144-3p	298
hsa-miR-5100	502
hsa-miR-6865-5p	351
hsa-miR-501-5p	615
hsa-miR-5196-5p	1309
hsa-miR-2277-3p	154
hsa-miR-1260a	567
hsa-miR-1909-3p	800
hsa-miR-4286	798
hsa-let-7g-3p	958
hsa-miR-1292-5p	132
hsa-miR-4784	831
hsa-miR-4304	53
hsa-miR-3928-5p	485
hsa-miR-4664-5p	469
hsa-miR-451a	40
hsa-miR-6069	164
hsa-miR-3614-3p	315
hsa-miR-3117-3p	225
hsa-miR-7850-5p	549
hsa-miR-3922-5p	543
hsa-miR-1908-3p	11
hsa-miR-6763-5p	833
hsa-miR-6071	819
hsa-miR-7977	1276

### *CPPED1* mRNA levels are decreased and *miR371a-5p* levels increased during spontaneous delivery

**Fig 2:**
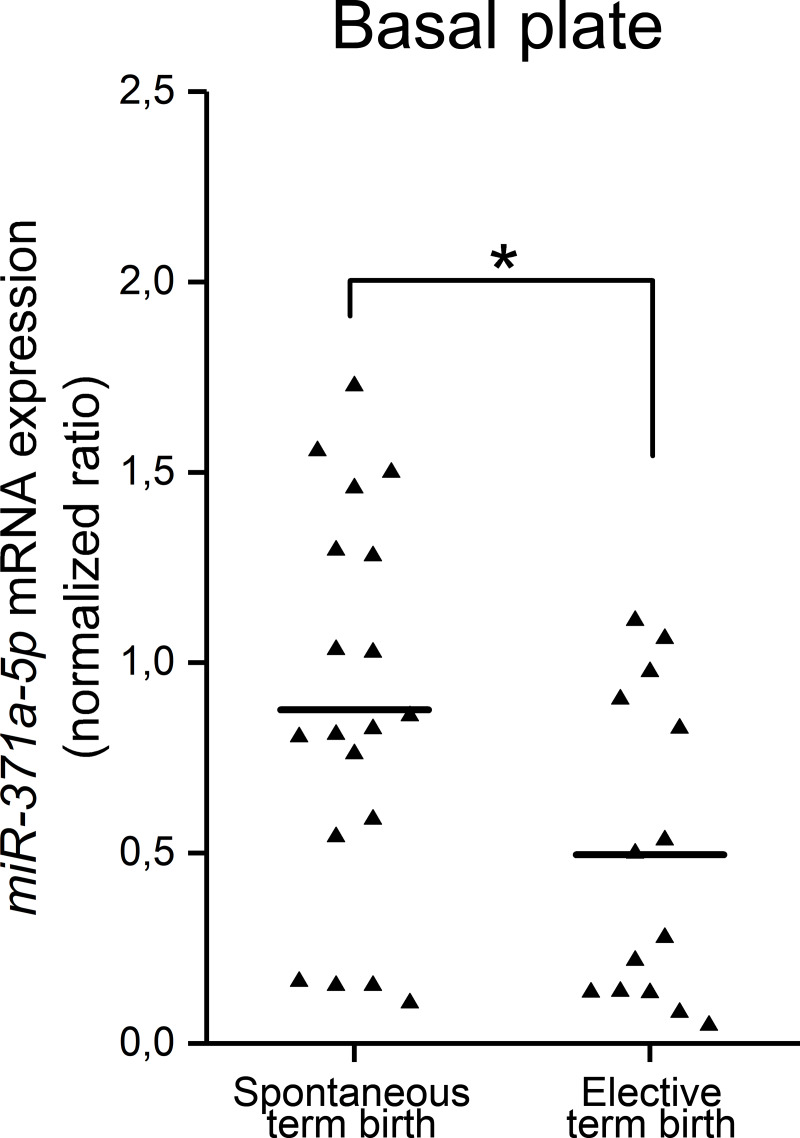
*Mir-371a-5p* levels in the basal plate of term placentas. Normalized expression levels of *miR-371a-5p* in placentas from spontaneous and elective term births. In the miRNAomic study, miRNAs isolated from human placenta after spontaneous term labor (*n* = 6) were compared with miRNAs obtained from elective deliveries (*n* = 6) without signs of labor. *Mir-371a-5p* was the second most upregulated miRNA at spontaneous term labor (fold-change = 2.6, *p* = 0.003). This result was confirmed in a larger number of placental samples (*n* = 19 for spontaneous term labor, *n* = 14 for elective term labor). During spontaneous term labor, *miR-371a5-p* levels significantly increased by 1.5-fold (*p* = 0.04). Differences were analyzed with nonparametric Mann–Whitney *U* test. Horizontal line denotes the median of each group. NS, not significant.

We previously found decreased amounts of CPPED1 in the basal and chorionic plates of the placenta at spontaneous term labor [23]. Levels of mRNA of *CPPED1* decreased accordingly. We extended this study to preterm labor and performed quantitative PCR (qPCR) analysis of *CPPED1* mRNA levels after spontaneous (*n* = 20) and elective caesarean (*n* = 33) preterm deliveries to determine whether *CPPED1* levels are associated with the initiation of labor or gestational age. We measured *CPPED1* mRNA levels separately for the basal and chorionic plates of the placenta (Fig 1). Moreover, we performed qPCR analysis of *miR-371a5-p* levels in placentas from spontaneous (*n* = 19) and elective (*n* = 14) term birth to confirm the miRNAomics finding. *miR-371a-5p* levels were determined only for the basal plate (Fig 2).

*CPPED1* mRNA levels decreased 1.5-fold (*p* < 0.001) in the chorionic plate of the placenta at spontaneous preterm birth. We did not observe this change in the basal plate of the placenta. At spontaneous term labor, *CPPED1* mRNA levels decreased in both the basal (1.2-fold, *p* = 0.04) and chorionic (1.5-fold, *p* < 0.001) plates (Fig 1). However, there was no difference in basal or chorionic plate levels of *CPPED1* mRNA between spontaneous preterm and spontaneous term deliveries, or between elective preterm and elective term deliveries. This suggests that *CPPED1* mRNA levels do not vary in accordance with gestational age; rather, they decrease at the onset of spontaneous labor regardless of gestational age. *MiR-371a-5p* mRNA levels increased 1.5-fold (*p* = 0.04) in the basal plate of the placenta during spontaneous term birth (Fig 2). The inversely correlated expression levels of *miR-371a-5p* and *CPPED1* suggest that miR-371a-5p binds to the 3′ UTR of *CPPED1* and downregulates CPPED1 levels.

Only the spontaneous and elective preterm birth samples included cases of preeclampsia and infection. Therefore, we subanalyzed the RT-qPCR results from these samples to investigate associations with pregnancy complications. In comparisons of spontaneous preterm and elective preterm cases, when we removed cases with preeclampsia and infection from all preterm groups (Fig 1), *CPPED1* expression levels were not affected. This suggests that *CPPED1* mRNA levels are not significantly affected by preeclampsia or intraamniotic infection.

### MiRNA seed regions of 3′ UTR of *CPPED1*

The 3′ UTR of *CPPED1* mRNA is 5087 base pairs (bp) long and predicted to be targeted by 175 different miRNAs, as determined by the MicroRNA Target Prediction and Functional Study Database (miRDB) [44,45]. Table 6 lists miRNAs with prediction scores of at least 80, which are considered putative or real binders to the 3′ UTR of *CPPED1* mRNA. MiR-371a-5p, of the miRNA:CPPED1 pair identified above, belongs to the placenta-specific miR-371-3 cluster and has three suggested binding sites in the 3′ UTR region of *CPPED1* mRNA, with a target prediction score of 84 (Table 6). None of the miRNAs listed in Table 6 belong to C14MC, the cluster from which miRNAs are expressed during the first trimester of pregnancy. However, miR-524-5p and miR-520d-5p are members of the late pregnancy–, placenta-specific cluster C19MC (Table 6). These findings suggest that early pregnancy–related miRNAs do not regulate *CPPED1* levels.

**Table 6:** Predicted miRNA binding sites in the 3′ UTR of CPPED1 mRNA. Putative miRNA binding sites in the 3′ UTR of *CPPED1* were analyzed with miRDB software [44,45]. Predicted targets with a prediction score of at least 80 were considered real. MiRNAs highlighted in orange belong to the placental chromosome 19 miRNA cluster (C19MC) or miR-371-3 cluster. In this study, miRNAs belonging to these clusters were checked for binding to the 3′ UTR of *CPPED1* mRNA.

Target rank	Target score	miRNA Name	Number of binding sites	Chromosomal location
1	100	hsa-miR-6867-5p	4	17q21.1
2	96	hsa-miR-4671-3p	2	1q42.2
3	95	hsa-miR-6801-5p	2	19q13.41
4	95	hsa-miR-10393-3p	2	15q21.1
5	94	hsa-miR-29b-2-5p	4	1q32.2
6	94	hsa-miR-1297	3	13q14.3
7	93	hsa-miR-568	3	3q13.31
8	93	hsa-miR-627-3p	5	15q15.1
9	90	hsa-miR-5696	3	2q11.2
10	88	hsa-miR-26a-5p	3	3p22.2
11	88	hsa-miR-524-5p	1	19q13.42
12	88	hsa-miR-26b-5p	3	2q35
13	88	hsa-miR-520d-5p	1	19q13.42
14	87	hsa-miR-580-3p	2	5p13.2
15	86	hsa-miR-6792-3p	4	19p13.2
16	86	hsa-miR-3128	1	2q31.2
17	86	hsa-miR-3934-5p	3	6p21.31
18	86	hsa-miR-4691-5p	4	11q13.2
19	85	hsa-miR-6733-3p	2	1p34.2
20	85	hsa-miR-4450	2	4q21.1
21	85	hsa-miR-4267	3	2q13
22	85	hsa-miR-205-3p	4	1q32.2
23	84	hsa-miR-4477b	1	9p11.2
24	84	hsa-miR-545-3p	3	Xq13.2
25	84	hsa-miR-4465	3	6q24.1
26	84	hsa-miR-371a-5p	3	19q13.42
27	84	hsa-miR-4775	5	2q33.3
28	83	hsa-miR-4277	3	5p15.33
29	83	hsa-miR-9985	2	Yp11.2
30	83	hsa-miR-3184-3p	3	17q11.2
31	83	hsa-miR-27a-3p	2	19p13.12
32	83	hsa-miR-27b-3p	2	9q22.32
33	82	hsa-miR-4729	2	17q22
34	82	hsa-miR-138-1-3p	2	3p21.32
35	81	hsa-miR-4777-5p	2	2q37.1
36	80	hsa-miR-4670-3p	1	9q22.31

### MiR-371a-5p and miR-520d-5p bind to 3′ UTR of *CPPED1*

**Fig 3:**
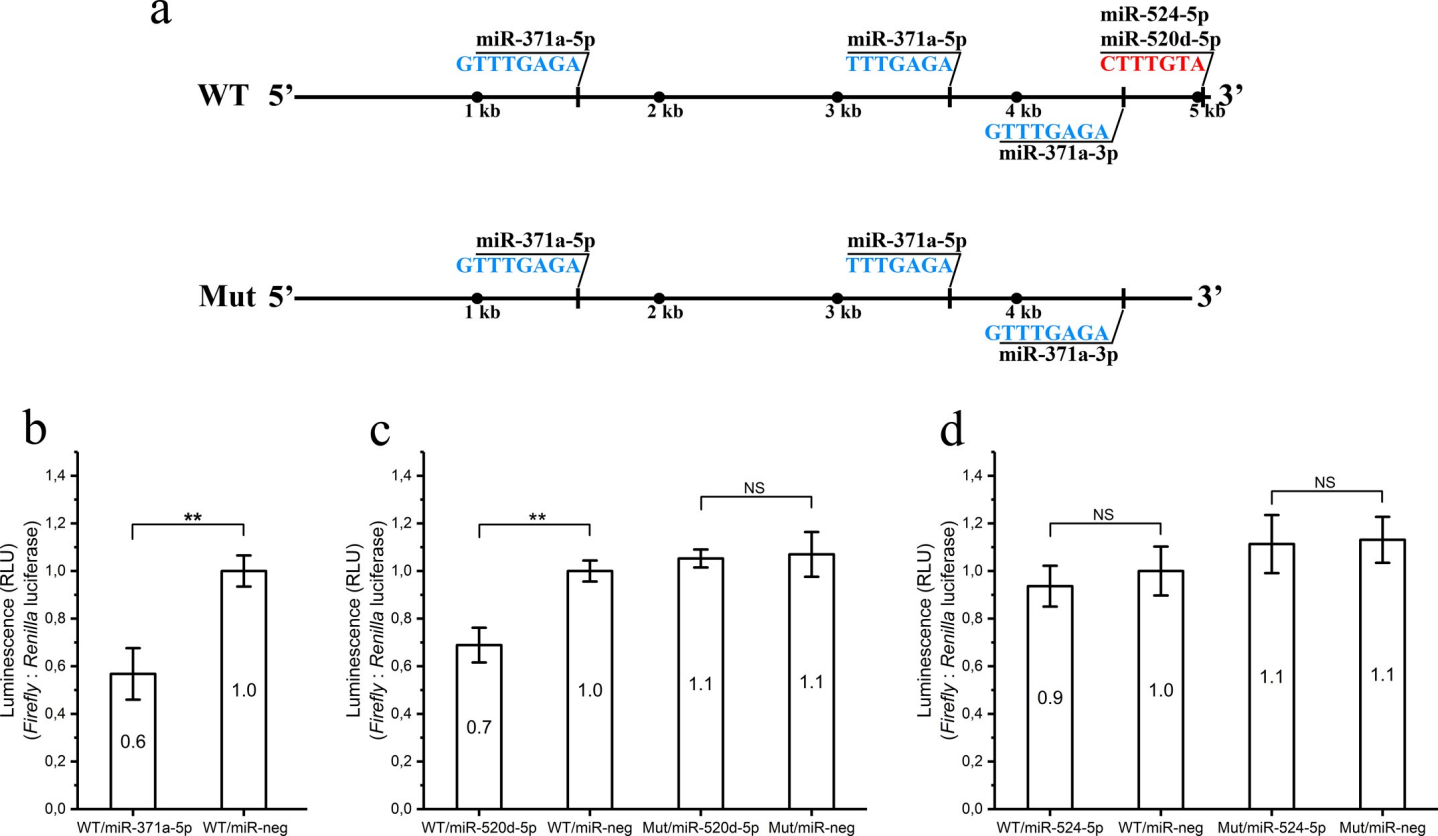
Binding of miRNAs in 3′ UTR of *CPPED1*. Seed locations of miRNAs analyzed for binding to the 3′ UTR of *CPPED1* by luciferase reporter assay (a). MiR-371a-5p has three seed locations, starting at 1602, 2033, and 4607 nucleotides. MiR-520d-5p and miR-524-5p share the same seed location in the end of 3′ UTR of *CPPED1*, which starts at nucleotide 5076. Seed locations are not to scale with respect to one another. For the luciferase assays, two variants of the 3′ UTR of *CPPED1* were constructed. One construct contained the full-length 3′ UTR (WT). The other construct (Mut) lacked the 33 terminal nucleotides containing the miRNA binding sites for miR-520d-5p and miR-524-5p. Black dots roughly mark every thousand kilobases (kb). Bottom part of the figure shows luciferase reporter–based assays to detect binding of miR-371a-5p (b), miR-520d-5p (c), and miR-524-5p (d) mimics to the 3′ UTR of *CPPED1* mRNA. WT or Mut constructs were cotransfected with miR371a-5p mimic (WT/miR-371a-5p) (b), miR-520d-5p mimic (WT/miR-520d-5p or Mut/miR-520d-5p) (c), or miR-524-5p mimic (WT/miR-524-5p or Mut/miR-524-5p) (d) into HEK-293T cells. For WT miR-neg and Mut miR-neg, the miRNA mimic negative control was cotransfected with WT or Mut constructs, respectively. Luciferase activities were measured by dual luciferase reporter assay, in which *Firefly* luciferase was the experimental reporter and *Renilla* luciferase the control reporter. Negative control values were set to 1.00 (WT/miR-neg), to which the rest of the samples were compared. Columns show the mean value of relative luciferase activity of six replicates; maximum and minimum values are also indicated. Statistically significant changes were determined by Mann–Whitney *U* test and are indicated by an asterisk (*p* < 0.05) or two asterisks (*p* < 0.01). NS, not significant.

To test if miR-371a-5p binds to the 3′ UTR of *CPPED1* mRNA, we used a dual-luciferase gene reporter assay to study post-transcriptional regulation in intact cells. In addition to miR-371a-5p of the miR-371-3 cluster, we also analyzed two C19MC miRNAs, miR-520d-5p and miR-524-5p (Table 6), for their effect on translation dependent upon the 3′ UTR of *CPPED1*. miR-371a-5p, miR-520d-5p, and miR-524-5p were the only miRNAs of the placental miRNA clusters predicted to bind to the 3′ UTR of CPPED1. Changes in *Firefly* luciferase levels (experimental reporter), with unchanged *Renilla* luciferase levels (control reporter), indicate binding of miRNA to the 3′ UTR. MiR-371a-5p has three putative binding sites (Table 6); these binding sites are distributed throughout the entire 3′ UTR of CPPED1 (Fig 3A). Both miR-520d-5p and miR-524-5p have one putative binding site (Table 6) close to the end of the 3′ UTR (Fig 3A).

Both whole (WT) and truncated (lacking the last 33 bps, Mut) 3′ UTRs of *CPPED1* mRNA were cloned into the pmiRGLO vector and transfected into the HEK-293T continuous cell line. The mutant construct lacked the seed regions for both miR-520d-5p and miR-524-5p (Fig 3A), while the WT and Mut constructs contained equivalent seed regions for miR-371a-5p (Fig 3A). The luciferase reporter assay revealed a significant decrease (*p* = 0.004) in the relative luciferase activity value of cells cotransfected with WT construct and miR-371a-5p mimic (WT/miR-371a-5p) (*n* = 6) compared to those transfected with WT construct and miRNA mimic negative control (WT/miR-neg) (*n* = 6) (Fig 3B). We observed a similar decrease when cells were cotransfected with WT construct and miR-520d-5p mimic (WT/miR-520d-5p) (*n* = 6, *p* = 0.004) compared to WT/miR-neg (*n* = 6) (Fig 3C). Importantly, the Mut construct abolished miR-520d-5p–mediated repression of luciferase activity. With the miR-524-5p mimic, we observed a slight decrease in the relative luciferase value compared to WT/miR-neg (*n* = 6, not significant) (Fig 3D). Although the luciferase experiment was done in HEK-293T cells and not in trophoblastic cells, the results indicate that miR-371a-5p of the miR-371-3 cluster and miR-520d-5p of C19MC bind to the 3′ UTR of CPPED1, suggesting negative regulation of *CPPED1* expression. However, whether this is the case in trophoblasts during pregnancy remains to be shown.

## Discussion

Little is known about the molecular mechanisms associated with onset of labor. It is important to have a better understanding of the pathophysiology and biochemistry of the placenta to fully understand pregnancy complications and the pathways leading to preterm and term deliveries. Similar to placenta-specific proteins such as pregnancy-specific glycoproteins (PSGs), placenta-specific protein 1 (PLAC1), and growth hormones of both maternal and fetal origin, miRNAs appear to have roles in the maintenance of pregnancy. Several studies have reported that variations in concentrations of pregnancy-specific proteins and miRNAs, as well as of cell-free RNAs, may serve as biomarkers in preterm labor and other pregnancy complications [46–48].

In the present study, we characterized the miRNAomes of placentas obtained after spontaneous and elective term deliveries. Subsequent comparative miRNAomic investigations identified several variations in miRNA expression levels associated with spontaneous labor. The genes encoding some of these miRNAs reside in C14MC and the miR371-3 cluster, which are known to be mostly placenta-specific miRNA clusters. None of the identified miRNAs belonged to C19MC, the identified third placenta-specific miRNA cluster. During pregnancy, levels of C14MC miRNAs decrease from the first to the third trimester. By contrast, C19MC miRNA levels can increase as much as 100-fold from the first to the third trimester of pregnancy [10,18]. The mouse miR-290-295 cluster is the homologue of the human miR-371-3 cluster. In the third trimester, the miRNAs of miR-290-295 cluster become localized to the placenta and reach the highest expression levels at birth [49]. At the same time, very low expression of miR-290-295 cluster miRNAs are seen in the embryo. These findings suggest that miRNAs in both C19MC and the miR-371-3 cluster are regulators during late pregnancy.

We found that in the human placenta, the entire miR-371-3 cluster was significantly upregulated during spontaneous term labor. The role of the miR-371-3 cluster in the placenta and pregnancy is not clearly known, and further studies are needed to understand its role. By contrast, miRNAs of the miR-371-3 cluster have been shown to be needed to maintain the pluripotent state of cells, early embryonic development, germline development, and differentiation of stem cells [50,51]. The miRNAs of this cluster target inhibitors of the G1/S transition [52]. The miR-371-3 cluster is epigenetically silenced in adult somatic cells, whereas it is highly enriched in embryonic stem cells (ESCs) in humans [53].

Our current study revealed that levels of miR-371a-5p and CPPED1 protein are inversely correlated in the human placenta during spontaneous term labor. We found that miR-371a-5p targeted human placental CPPED1. MiR-371a-5p is a member of the placenta-specific miR-371-3 cluster. Altered expression of miR-371a-5p is associated with pregnancy complications. Gestational trophoblastic disease (GTD) includes a wide range of diseases that arise from abnormal proliferation and differentiation of trophoblasts; the most common form is known as a hydatidiform mole (HM), which may develop into malignant gestational trophoblastic neoplasia (GTN). MiR-371a-5p levels are upregulated in GTN, and elevated levels are associated with enhanced proliferation, differentiation, and invasion in choriocarcinoma cells [54]. Recurrent pregnancy loss is a condition in which apoptosis dominates trophoblastic growth, which results in early pregnancy termination. Expression of miR-371-5p and its target gene X-linked inhibitor of apoptosis protein (XIAP) are greatly decreased and associated with increased apoptosis leading to pregnancy loss [55]. These findings suggest that in uncomplicated pregnancies, miR-371a-5p levels increase toward the end of pregnancy and aberrant expression of miR-317a-5p, especially in early pregnancy, results in severe adverse outcomes.

In addition to miR-371-3 cluster miRNAs, there are putative binding sites for C19MC miRNAs in the 3′ UTR region of CPPED1. We tested the effect of two miRNAs, miR-520d-5p and miR-524-5p of the C19MC, on the expression of *CPPED1* and showed that miR-520d-5p binds to the 3′ UTR of CPPED1. Earlier studies showed that miR-520d is a placenta-specific miRNA and is secreted into the maternal circulation [9,56]. Targets of miR-520d-5p are related to cancer progression. In colorectal cancer, reduced levels of miR-520d-5p lead to cancer progression by enhancing proliferation, migration, and invasion of cancer cells [57]. By contrast, higher levels of miR-520d-5p restore E-cadherin expression, which results in reduced cancer cell motility and invasiveness. Consequently, higher miR-520d-5p levels are associated with higher survival rates in cancer patients [58].

Specific cancer types and the placenta share certain properties, such as a high proliferation rate, invasion of tissue, and modulation of the host immune response [59]. Previous findings have indicated that expression of miR-371a-5p or miR-520d-5p affects cancer phenotypes, including cell proliferation, cell motility, and, ultimately, cancer progression. We found that miR-371a-5p levels were upregulated in spontaneous term delivery compared to elective caesarean section delivery without signs of labor. Thus, during spontaneous labor, upregulation of miRNA-371a-5p in the human placenta could suppress cell functions related to immune tolerance in a manner similar to what is observed during cancer progression [58]. We propose that this may be mediated in part by decreasing levels of the phosphatase CPPED1. In line with this, siRNA silencing of *CPPED1* in cultured trophoblasts affects pathways involved in blood vessel development and cytokine activity [23], which is also seen in tumors and trophoblastic invasion [60,61]. Our pathway analysis of the validated targets of miRNAs that we identified in our investigation of the placental miRNAome was enriched for several cancer-related pathways. This provides further evidence that miRNAs associated with spontaneous labor are also involved in carcinogenesis. Additionally, the PI3K-AKT and FOXO signalling pathways were among the enriched pathways. AKT regulates the activity of FOXO, which affects genes involved in the cell cycle, apoptosis, and the immune system [62]. FOXOs also contribute to preeclampsia, fetal growth restriction, and spontaneous labor [63,64].

In conclusion, our results suggest that high expression levels of miR-371a-5p are associated with decreased levels of CPPED1. In our previous study, high levels of CPPED1 were associated with a long duration of pregnancy whereas low expression and low levels of CPPED1 signaled spontaneous labor, with possible involvement of CPPED1 phosphatase activity [23]. Earlier studies also showed that high levels of miR-371a-5 and low levels of CPPED1 are associated with striking functional effects in cancer tissue [24,54]. The mechanism by which placental miR-371a-5p and CPPED1 promote labor is a topic for future investigations.
